# Collagenous Tissues upon Lithium Treatment: A Quantitative Ultrastructural Study

**DOI:** 10.1100/tsw.2004.119

**Published:** 2004-08-11

**Authors:** Margaret Tzaphlidou

**Affiliations:** Laboratory of Medical Physics, Medical School, Ioannina University, 45110 Ioannina, Greece

**Keywords:** electron microscopy, ultrastructure, electron-optical data, image analysis, collagen, lithium, skin, liver, bone, cranial arachnoid and dura mater, aorta

## Abstract

In this review, the influence of lithium treatment in mouse, rat, and rabbit skin, liver, bone, and aorta, as well as arachnoid and dura mater collagen fibrils, is examined using electron microscopy and image processing. Structural changes (fibril architecture and diameter) are detected at the ultrastructural level in specimens from all lithium-treated tissues. The overall collagen fibril architecture is disturbed as compared with specimens from normal species. The mean diameter values of treated collagen fibrils are significantly smaller than those from controls in all tissues examined. The banding patterns of fibrils are normal in all cases. Measurements by a computerized method of measuring axial periodicity of fibrils indicate no effect of lithium on this parameter. Computer analysis shows no differences in charged amino acid composition between lithium-treated and -untreated samples. Under the present experimental conditions, lithium can induce permanent structural collagen alterations.

## INTRODUCTION

Lithium has been used as a treatment for various psychiatric and somatic illnesses for more than 50 years[1]. A considerable amount of data shows lithium to be an effective agent in the prophylaxis of major depressive disorders[2,3,4,5,6], although the evidence is less clear than in the case of bipolar disorder. Lithium is generally considered to be the gold standard for maintenance therapy in bipolar disorder[7,8,9,10], reducing the risk of relapse too[11]. There is also strong evidence for lithium's role in augmenting antidepressant therapy[12]. In addition, long-term lithium treatment lowers suicide risk in major affective disorders[13,14].

It has been proposed that lithium's therapeutic effect on mental diseases is via the stimulation of the immune system. Genetic factors also appear to play a significant role in the outcome of long-term lithium treatment[15,16,17,18]. Lithium appears to play an especially important role during the early fetal development as evidenced by the high lithium content of the embryo during the early gestational period. The biochemical mechanisms of the action of lithium are multifactorial and intercorrelated with the functions of several enzymes, hormones, and vitamins, as well as with growth and transforming factors[19].

Although lithium remains the standard against which all proposed alternatives are compared[20], treatment with lithium induces functional and/or structural disturbances in various tissues and organs. This review assesses the side effects of lithium in a number of tissues/organs and investigates the impact of collagen on these effects.

## LITHIUM EFFECTS ON VARIOUS TISSUES AND ORGANS

### Skin

Lithium may produce several dermatological side effects[21,22]. Acneiform eruption, psoriasis, folliculitis, and maculopapular eruption have been described as adverse reactions to lithium therapy[23]. The predominant cutaneous side effect of lithium is the exacerbation or aggravation of psoriasis[24,25]. Pathological findings of neutrophilic infiltration have also been described[22]. To our knowledge, however, the effects of lithium on collagen, which is one of the major components of skin, have not been extensively reported in the literature.

### *Abnormalities of Skin Collagen Fibril Structure Due to Lithium Treatment*

Lithium chloride treatment produces severe alterations in the structure of skin collagen fibrils. In thin-sectioned fibrils, cut longitudinally, the overall architecture is disturbed. In cross-sections, lithium has a dramatic effect on fibril diameter as well as on its profile.

#### The Role of Lithium Dose

The role of lithium dose on possible side effects of skin collagen induced by lithium were studied by using male Swiss Albino mice 5 weeks of age. Animals were injected (intraperitonealy) once a day for 30 consecutive days and were kept in groups of four; they were sacrificed 1 day, 1, 2, and 6 months after the last injection. The reasons for choosing these parameters for lithium administration are given elsewhere[26]. This was the longest period of treatment for all tissues examined, as the usual duration of treatment in animal studies is between 15–30 days. Mice were treated with four different lithium doses: 6, 1.5, 0.7, or 0.3 meq Li/kg of body weight. They could not withstand, without apparent difficulty, lithium levels greater than 6 meq Li/kg. Most mice showed muscular weakness after a few injections at these levels. The 1.5-meq-Li/kg dose is greater than that used in the human patient[19]. To maximize possible lithium effects on collagen structure, it is necessary to use as high a dose as possible. The 0.7-meq-Li/kg dose is closer to the therapeutic one, while the 0.3-meq-Li/kg dose is within or even below the therapeutic limits. Age-matched animals without injections served as controls. The procedures for preparation of specimens for electron microscopy and the morphometric analysis are described in a previous publication[27].

**Fibril architecture** — For doses of 6–0.7 meq Li/kg, the overall collagen fibril architecture is dramatically affected. The effects are more pronounced when the dosage is higher. The characteristic parallel packing of fibrils is usually replaced by a random arrangement (Fig. 1b–d). In some regions, the characteristic parallel packing of normal fibrils shown in Fig. 1(a) is lost completely (Fig. 1b,d), whereas in others, it is preserved for a small part of the area (Fig. 1c). Fibrils showing twisting, variations in thickness, and forming a “hairpin” loop are apparent[28,29]. Spiraled aggregates of small fibrils resulting

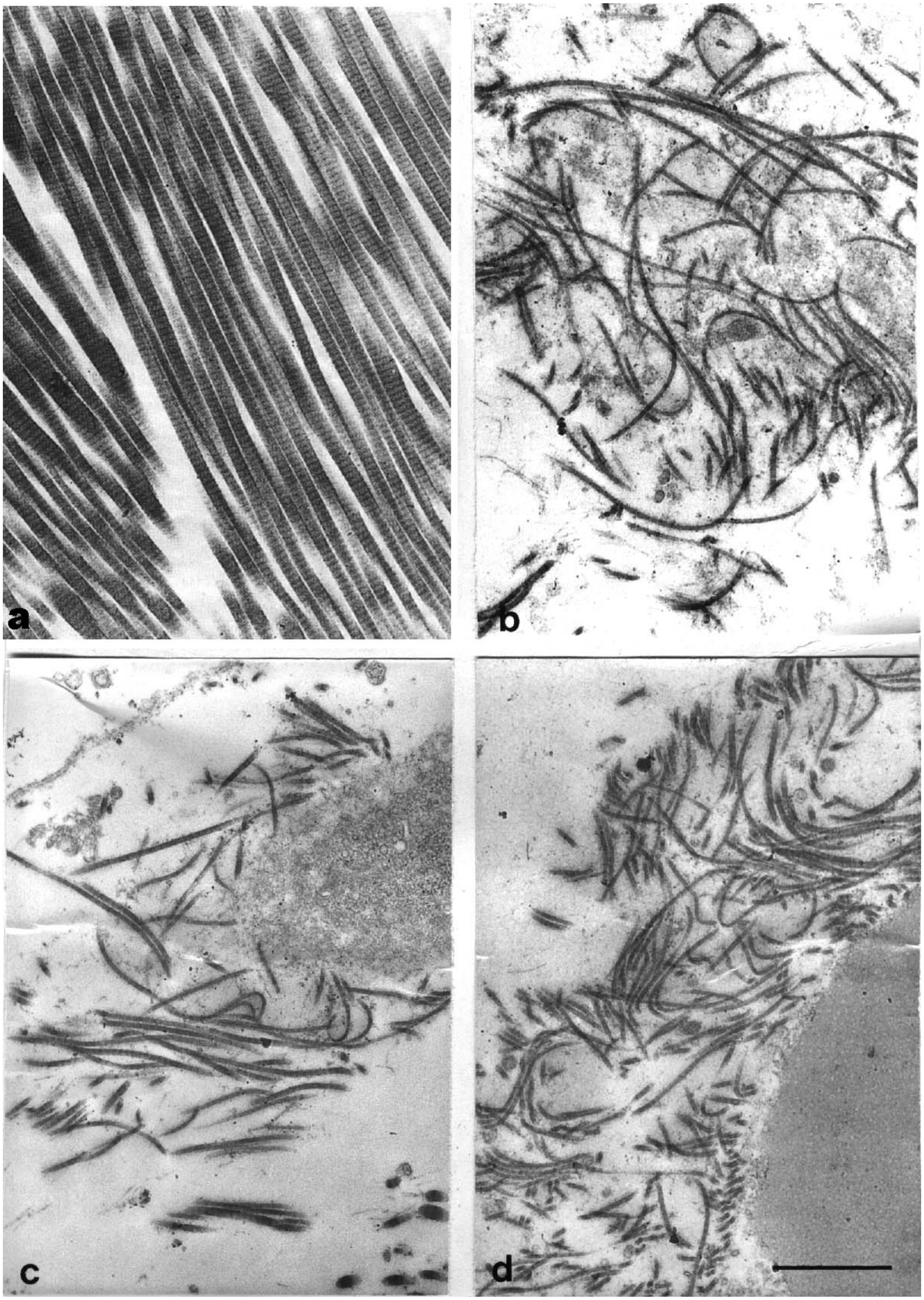

**FIGURE 1.** Electron micrographs of mouse skin collagen treated with 6 meq Li/kg for 30 consecutive days (b–d) demonstrating fibrils in disarray. In some regions, the characteristic parallel packing of fibrils is lost completely (b,d), whereas in others, it is preserved in a small part of the area (c). Animals were sacrificed (b) 1 day, (c) 2 months, and (d) 6 months after the last lithium injection. (a) Normal mouse skin fibrils. Bar = 1.0 µm.

in abnormally thick fibrils are very frequently detected (Fig. 2a, indicated by arrows), which are absent in normal fibrils (Fig. 2b). These abnormalities are observed from the first stage, i.e., when animals are sacrificed 1 day after the last lithium injection, and are more pronounced at later stages, i.e., 6 months after the last lithium injection. With a dose of 0.3 meq Li/kg, in most areas the normal parallel packing of fibrils is preserved. However, there are areas where the twisting of fibrils seen in higher doses is apparent[29].

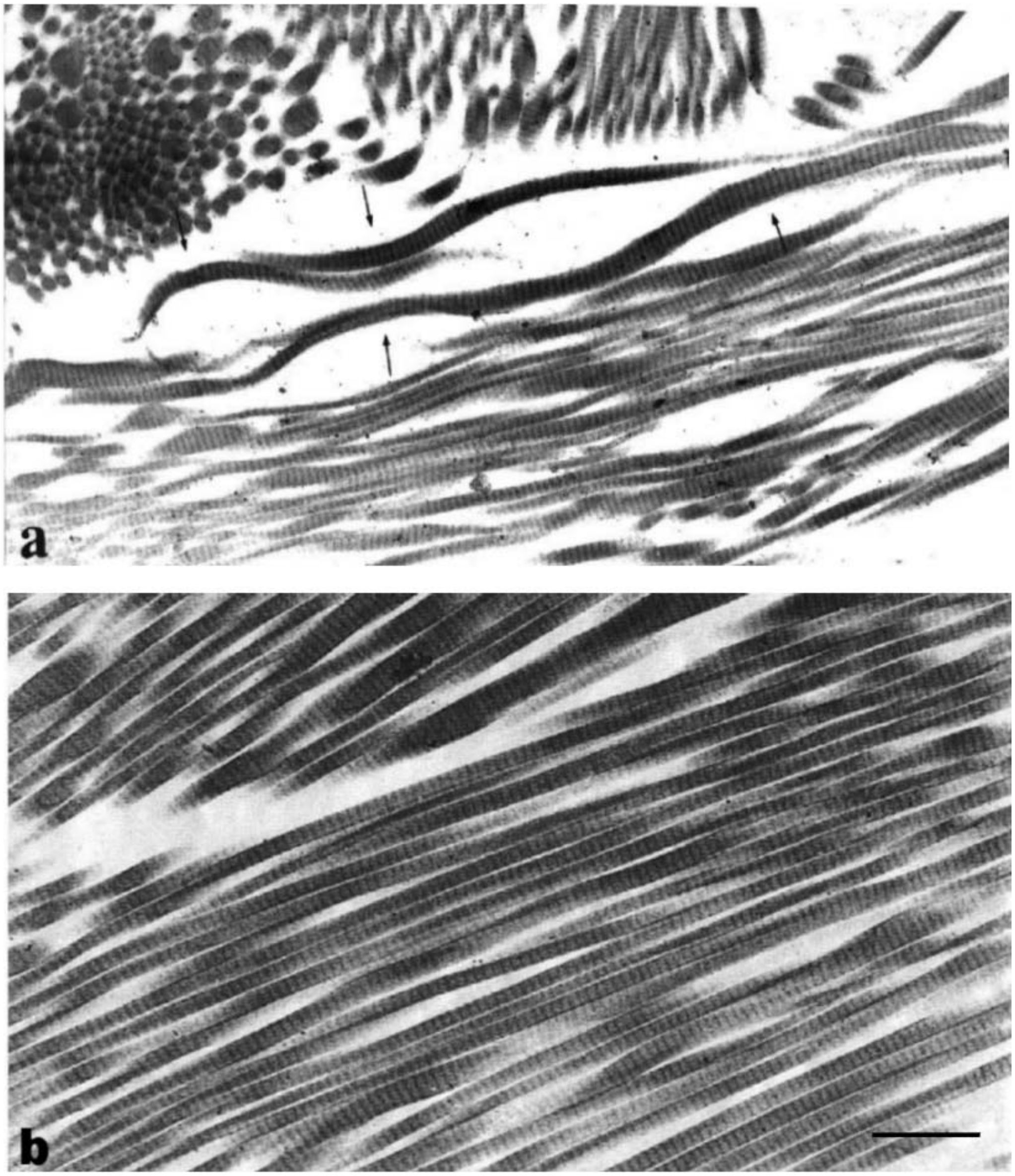

**FIGURE 2.** Twisted skin collagen fibrils from mice treated with 6 meq Li/kg for 30 consecutive days (a). Some abnormally thick fibrils are present (indicated with arrows), which are due to spiraled aggregates of smaller fibrils. Animals were sacrificed 1 month after the last lithium injection. (b) Normal fibrils for comparison. Bar = 0.5 µm.

**Fibril diameter** — Fibrils from lithium-treated animals at doses of 6–0.7 meq Li/kg are significantly ($p$ = 0) smaller in their diameter compared to normal with a high variability in width[28,29]. Many abnormal large fibrils, “clusters”, are distributed among the cross-sections with an irregular outline deviated from an ideal circular or elliptical profile. They are more prominent at late stages. As the dose becomes higher, the population of the clusters is increased. They constitute approximately 10 to 22% of the whole population depending on the dose. With the lowest dose used, i.e., 0.3 meq Li/kg, such clusters are not observed[29] and there is no high variability in fibril width.

## The Effect of Treatment Duration

At a short treatment period of 7 days with lithium doses 0.7 and 1.5 meq Li/kg, a high level of skin collagen fibril disorganization is observed while fibril diameter is less affected, indicating that this parameter is dependent on the duration of lithium treatment[29].

With both doses, although there are regions where the packing of fibrils is similar to normal, many fibrils have an anarchic arrangement. Fibrils in a bundle, instead of being in register with the rest of the fibrils, form a very characteristic “hairpin loop”. The effect of twisting is very pronounced. Variations in fibril thickness are not apparent.

Fibrils are smaller than the control. However, the decrease in mean diameter is greater at prolonged times after treatment, i.e., when the animals were not sacrificed after the end of the experimental period[29]. Clusters with abnormal fibrils found in longer period treatments are not observed.

The most notable feature of the results concerning the fibril diameter is that this parameter is dependent on lithium treatment duration. As the time of treatment increases to 15 or 21 consecutive days, collagen fibrils become smaller ($p = 0$). In all time points, lithium mean diameter values differ significantly ($p = 0$) than the corresponding normal ones.

The dose of 0.3 meq Li/kg, at short period treatment of 7 days, has a slight effect on fibril diameter compared to normal ($p = 0.13$).

## The Experimental Species

To seek information as to whether or not the observed structural effects of lithium on skin collagen fibrils are dependent on the experimental species, in addition to mice, Wistar rats at the same age and rabbits 7 months of age were used. Animals were injected for 30 consecutive days with a lithium chloride dose of 1.5 meq Li/kg.

Severe alterations in rat and rabbit skin collagen fibrils were detected similar to those observed in mice[27]. In most areas, the arrangement of fibrils is very disorganized (Fig. 3a,b) with helical twisting (Fig. 3c). As in mice, rat and rabbit lithium-treated fibrils are significantly ($p < 0.01$) smaller in diameter than those found in the control with a high variability in width. Abnormally large fibrils are also present throughout, distributed among others with extremely small diameters. These abnormalities are present in all stages.

In conclusion, these results show that lithium, under the present experimental conditions, can induce similar structural skin collagen alterations to all experimental species used.

## The Effect of Age

Male mice and rats, 5 weeks and 1 year old, were used to study the role of age on lithium side effects on skin collagen. Animals were treated with lithium as already described. They were sacrificed 1 day and 6 months after the end of treatment. Age-matched animals that were given no injections served as controls.

Skin collagen fibrils from young and adult mice and rats treated with lithium do not assemble normally. When studied at the ultrastructural level, fibrils in marked disarray are observed. In general, on lithium treatment, the overall structural architecture of collagen fibrils, at both ages and experimental species, is disturbed (Fig. 4a,b).

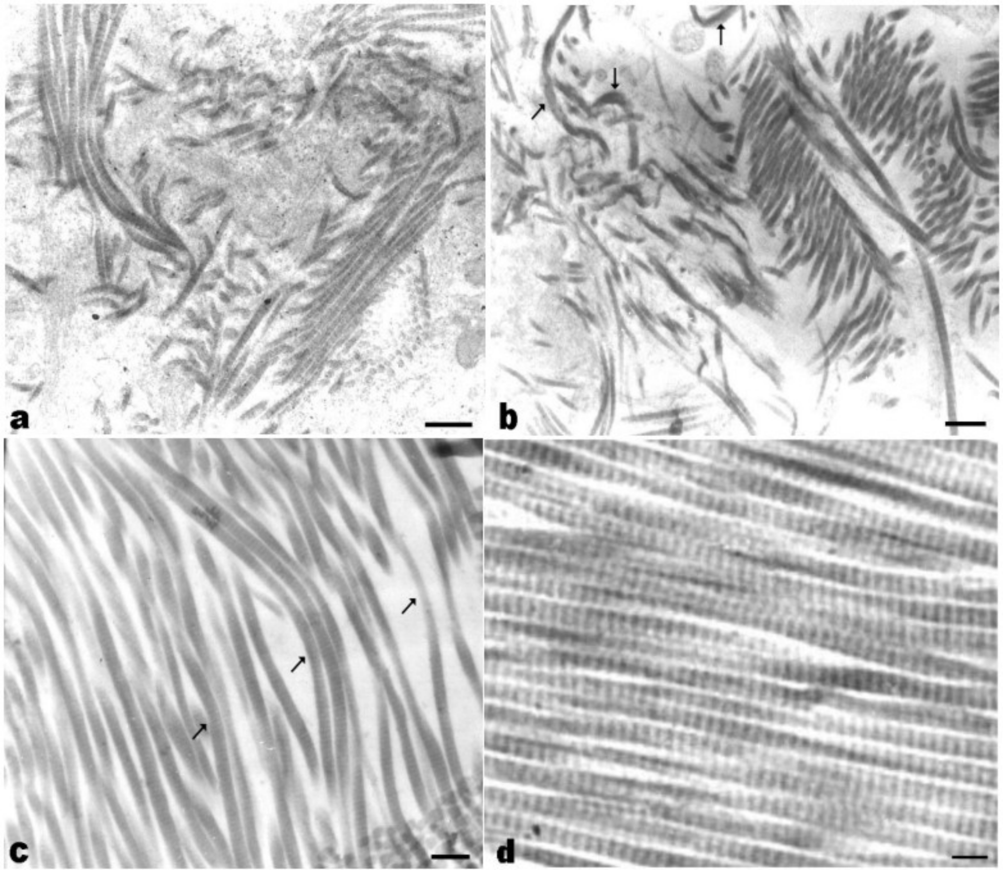

**FIGURE 3.** Effects of lithium on rabbit skin collagen. (a,b) The arrangement of fibrils is very disorganized. In some arrays, fibrils change their orientation forming a "hairpin loop" (b, shown with arrows). (c) Helical twisting (indicated with arrows) is also apparent. Animals were injected with 1.5 meq Li/kg for 30 consecutive days and were sacrificed 1 day, 2, and 6 months after the last lithium injection, respectively. (d) Skin fibrils packed in roughly parallel array from normal rabbits. Bar = 0.9 µm.

Lithium has also a dramatic effect on skin collagen fibril diameter. Throughout, there is a significant difference ($p = 0$) in the mean diameter between the treated and control subjects. In both experimental species studied, lithium has the same effect on fibril diameter at both ages. When viewed in cross-sections, fibrils treated with lithium have a high variability in width, in contrast to normal (Fig. 4c,d). Many clusters of abnormal fibrils (Fig. 4c, indicated by arrows) are found throughout.

## Liver

Lithium induces hepatic dysfunction owing to its toxicity[30]. It acts on liver glycogen metabolism *in vivo* in at least two different ways: one related to changes in insulinemia and the other related to the direct action of lithium on the activity of some key enzymes of liver glucose metabolism[31]. It inhibits the expression and secretion of insulin-like growth factor-binding protein-1[32]. It has been reported that lithium therapy may prove effective in improving the impaired antioxidant status during diabetes[33].

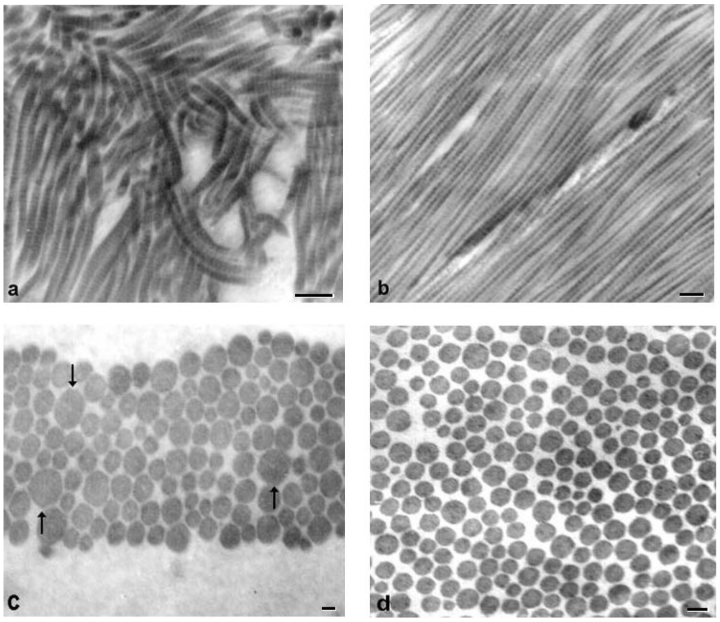

**FIGURE 4.** Electron micrographs of rat skin collagen: (a,c) treated with 1.5 meq Li/kg for 30 consecutive days. Animals were sacrificed 6 months after the last lithium injection. In (a) fibrils in disarray are shown while in (c) collagen fibrils with considerable variation in diameter are present. Such fibrils are significantly smaller than the untreated fibrils. Abnormally large fibrils (indicated by arrows) with an irregular elliptical profile are also present. (b) Normal skin fibrils packed in roughly parallel array. (d) Control fibril diameters for comparison. Bar = 0.6 µm.

### *Abnormalities of Liver Collagen Fibril Structure Due to Lithium Treatment*

After lithium treatment for 30 consecutive days with injections each corresponding to 1.5 meq Li/kg, similar observations are obtained for both experimental species studied, i.e., rats (5 weeks and 1 year old) and rabbits (7 months old). Although there are regions where the packing of fibrils tend to be similar to normal, many fibrils have an anarchic arrangement. In addition, fibrils change their orientation[27].

Liver collagen fibrils from rats and rabbits treated with lithium are significantly ($p < 0.01$) smaller in their diameter compared to normal with a high variability in width. Clusters (aggregates of smaller fibrils) with an irregular outline are observed at prolonged time after treatment, i.e., 6 and 12 months after the last lithium injection[27]. The population of these abnormally large fibrils, constituting about 5% of the whole population in rats and 2% in rabbits, is lower than in skin.

A point worth noting is that even 12 months after lithium treatment, collagen abnormalities are present. The time required, if any, for recovery after treatment cannot be suggested here. In the case of skin, the predominance of type I collagen during development may suggest that this collagen by itself may play a role in the organization of fibrils in this case.

## Bone

Lithium is known to accumulate in bone. It may effect calcium metabolism and alter parathyroid physiology by causing hypersecretion of parathyroid hormone, PTH[34,35]. Studies of patients on lithium reveal a clear alteration in PTH dynamics[36]. Short- or long-term treatment with lithium has no apparent effect on bone density[37,38] although an increase in bone turnover is associated with this treatment. According to Leutgeb[39], the effect of lithium on bone should be considered only in osteomalacia and severe osteoporosis.

Holak and Raisz[40], studying the effects of lithium on bone metabolism, demonstrated that lithium does not inhibit the incorporation of labeled proline in collagen and noncollagen protein. Collagen types synthesized by murine bone marrow cells and the effect of lithium chloride on collagen biosynthesis have been studied *in vitro* experiments[41]. Lithium treatment did not affect the types of collagen synthesized, although the relative proportions of collagen types may differ from controls. However, lithium does have an effect on the appearance of some noncollagenous components in the cell culture media.

### Abnormalities of Bone Collagen Fibril Structure Induced by Lithium

Male Swiss Albino mice 5 weeks of age were injected with lithium chloride (1.5 meq Li/kg) once a day for 30 consecutive days. Structural alterations in bone collagen fibrils treated with lithium are detected at the ultrastructural level. The abnormalities are not observed immediately after the end of treatment, but become prominent at prolonged time after treatment (2 and 6 months).

When mice are sacrificed 1 day after the last lithium injection, the packing of fibrils resembles normal, while at later stages marked disorganization in their packing as well as irregularity of shape of fibrils is seen[42].

At the early stage, 1 day after the end of the experimental period, the mean fibril diameter value (44.2 ± 10.7 nm) is close to normal (44.0 ± 7.4 nm). In the contrary, at later stages (i.e., 2 and 6 months after treatment), mean diameters are significantly ($p < 0.001$) smaller, 42.6 ± 9.8 and 41.2 ± 0.8 nm, respectively, than those found in the control, 45.0 ± 6.3 and 46.3 ± 7.8 nm, respectively. In the last cases, occasionally, clusters of abnormal fibrils are present constituting a very small percentage of the whole population.

## Brain

There are a great number of reports on the actions of lithium in the brain. Chronic lithium treatment affects rat brain[43]. It significantly inhibits brain glycogen synthase kinase-3 *in vivo* at concentrations relevant for the treatment of bipolar disorder[44]. Also, the central nucleus of the amygdala plays an important role in the neural framework that is responsible for the mood-stabilizing effect of lithium[45,46,47]. Lines of evidence[48] have led to the suggestion that chronic lithium regulates transcriptional factors, which in turn may modulate the expression of a variety of genes that compensate for aberrant signaling associated with the pathophysiology of bipolar disorders. The involvement of alterations at the level of gene expression in the mechanisms underlying the therapeutic effects of lithium have also been reported by Wang et al.[49] and Hua et al.[50]. Chang and Jones[51] suggest that a major therapeutic effect of lithium is to attenuate brain phospholipase A2 activity involved in signal transduction. In the contrary, Weerasinghe et al.[52], after chronical administration of lithium chloride to rats, concluded that lithium does not affect the fractional phosphorylation of brain cytosolic phospholipase A2, while reducing its net protein level. Lithium alters levels of key membrane phospholipids and appears to affect the balance between inhibitory and excitatory amino acids in rat brain[53,54]. Other data point to a link between the therapeutic efficacy of lithium and the response of 5-hydroxytryptamine-1 [5-HT1][55] and dopamine D2[56] receptors. In a rat model of depression, lithium treatment altered the brain concentrations of neurotrophic factors[57]. Although animal studies have demonstrated neuroprotective effects of lithium[58], it remains to be established whether lithium treatment protects against possible cell damage in the same manner as it protects against recurrences of bipolar and neurodegenerative disorders.

## Lithium Effects on Rat Cranial Arachnoid and Dura Mater Collagen

The effects of lithium chloride (1.5 meq Li/kg of body weight) on rat cranial arachnoid and dura mater collagen fibrils were examined by electron microscopy and image analysis. Animals were sacrificed 1 day, 2, 6, and 12 months after the end of a 30 consecutive days experimental period. No longer-term lithium treatment was investigated, as in studies concerning lithium effects in rat brain, the usual duration of treatment is between 15–30 days[47,49,53,55]. In all cases investigated, severe structural abnormalities in collagen fibrils are found[26].

These abnormalities consist of a marked decrease ($p < 0.001$) in mean diameter of fibrils from lithium-treated rats compared with normal as well as of a high variability in fibril width (Fig. 5a–c). Abnormally large fibrils (clusters) detected in other tissues are also present here. This feature becomes very prominent at the two later stages. The population of these fibrils is approximately 15% of the whole. About 2% of these fibrils are even larger. They consist of less-packed fibrils giving an externally lobulated cross-sectional profile (Fig. 5b,c, indicated by arrows).

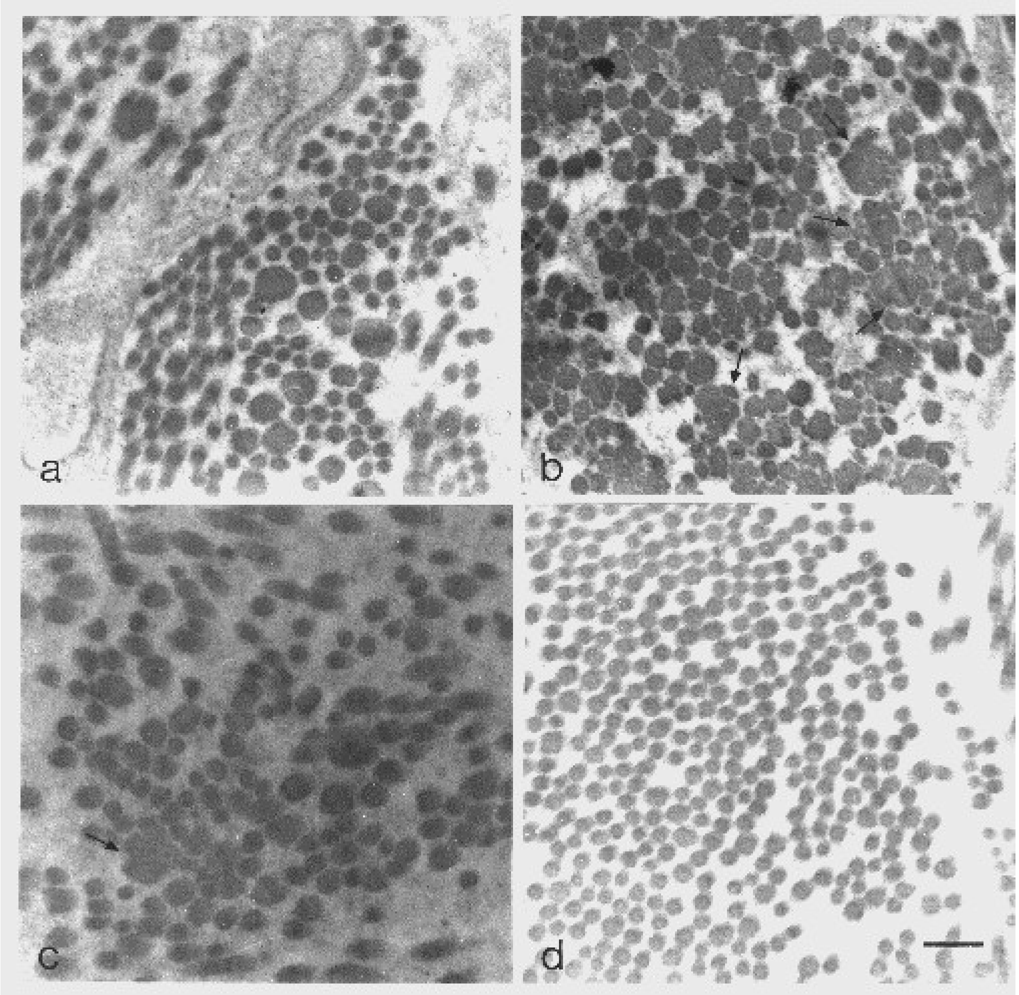

**FIGURE 5**. Ultrastructure of collagen fibrils seen in cross-sections. In (a–c) the animals were injected for 30 consecutive days with 1.5 meq Li/kg and were sacrificed 6 months (a,b) and 12 months (c) after the last injection. The largest fibrils (indicated by arrows) are highly irregular with lobulated profile. These are compared with control fibril diameters (d). Bar = 0.3 µm.

Severe disorganization in the packing of the fibrils is a persistent observation in all stages, i.e., the time that the animals were sacrificed after the last lithium injection.

It is important to recognize that these abnormal structures are present in rat tissues after a relatively short period of lithium treatment (30 days). A point worth noting is that they are progressive with time after treatment.

## Aorta

Long-term lithium treatment is linked with an increase in endothelium-dependent relaxation induced by acetylcholine in rat aorta[59,60]; it also prevents endothelial alterations of the rabbit aorta induced by catecholamines[61]. In addition, an enhancement to vascular contractions by lithium stimulated by angiotensin II has been demonstrated[62]. In a rat ischemia/reperfusion model, lithium treatment decreased the number of neurons showing DNA damage in the ischemic brain[63].

### *Effect of Lithium Administration on Collagen of the Rat Thoracic Descending Aorta*

With a lithium dose of 1.5 meq Li/kg, after 30 days of treatment, structural alterations in rat thoracic descending aorta collagen fibrils are detected at the ultrastructural level[64]. These abnormalities in longitudinal sections are similar to those observed in other tissues. Results regarding the effect of lithium on fibril diameter resemble those on bone collagen. Fibril diameter is not statistically significantly ($p < 0.1$) altered immediately after the end of treatment; mean diameter fibril values for lithium treated and untreated fibrils are 80.1 ± 15.5 and 81.7 ± 7.6 nm, respectively. However, it becomes enhanced ($p < 0.05$) at prolonged times after treatment, i.e., 2 and 12 months; mean diameter values for treated fibrils are 77.3 ± 16.6 and 78.6 ± 12.8 nm, respectively, while the corresponding ones for untreated fibrils are 83.3 ± 5.9 and 80.1 ± 6.4 nm.

## EFFECT OF LITHIUM ON COLLAGEN POSITIVE STAINING PATTERN

The D-periodic band pattern of collagen fibrils arises because the long rod-like molecules occur in near-parallel array, regularly staggered with respect to one another by a constant axial displacement D or integral multiples thereof[65]. Following positive staining with solutions of heavy metal salts, up to 12 staining bands of differing widths per D-period can be distinguished[66]. This characteristic fibril staining pattern, as seen in the electron microscope, appears to be common to collagen from a wide variety of sources.

Tzaphlidou[67] reported that the staining pattern may be used as a tool to seek information about the changes produced in abnormal collagen by its deviant molecular or fibril architecture. Providing that this information is correlated with that from other methods, the staining pattern may serve as an additional marker for the determination of the factors responsible for collagen abnormalities brought about by a treatment such as lithium or a clinical disorder.

### Positive Staining Patterns of Lithium-Treated Collagen Fibrils

All collagen fibrils from mice, rats, and rabbits treated with lithium in all tissues referred in this study, including the parts where disorganization occurs, retain the normal banding periodicity. Fig. 6 compares a normal rat liver (a) collagen banding pattern with a pattern from the respective lithium-treated material (b). As seen, the classical D-periodicity can be recognized. The band pattern is well preserved, i.e., the 12 staining bands per period are readily observed. Thus, we may conclude that lithium does not disturb the normal axial relationships between molecules in a collagen fibril from the experimental species and tissues discussed in this review.

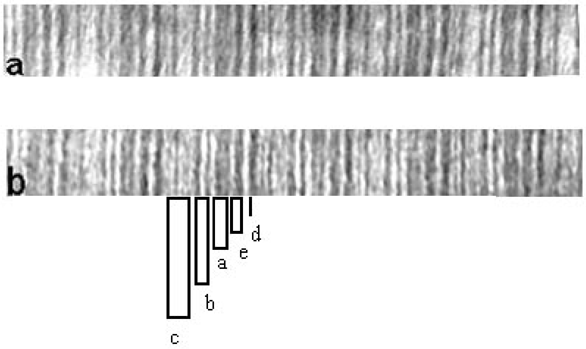

**FIGURE 6.** Positive staining patterns from sections of rabbit liver collagen. The sections were doubly stained with phosphotungstic acid and uranyl acetate. In (a), the staining pattern is from collagen treated with 1.5 meq Li/kg. Animals were sacrificed 1 day after the end of treatment. (b) Normal staining pattern. Both patterns are at the same magnification. Labelling of the bands follows the notation of Hodge and Schmitt[68]. ×330,000.

## Computer Analyses of Staining Patterns

Computer analyses were based on the assumption that collagen fibrils can be treated as "one-dimensional objects" in which only variations in image intensity in one direction, i.e., along the fibril axis, need to be considered. These axial variations are, moreover, periodic.

Using a least-squares analysis, Tzaphlidou and Kounadi[69] obtained the mean intensity distribution for a D-period for mice skin specimens lithium treated and untreated. Comparing the two sets of data quantitatively by the Kolmogorov-Smirnov (K-S) two-sample test, they found out that the treated and untreated positive staining patterns are the same at the confidence level 0.001. As the staining bands have long been thought to be due to the uptake of the staining ions on charged amino acid side groups along the collagen molecules[68,70], the agreement between the two patterns may indicate that lithium has zero or only a small effect on modifying the charge distribution along the mouse skin collagen fibril. Also, this may indicate that the charge distribution is not altered on lithium treatment. As the exact position of molecules when they assemble together to form fibrils is mainly controlled by the electrostatic interactions, it makes it easy to understand the significance of this observation.

Computer-aided analysis of electron-optical images from untreated and lithium-treated fibrils was also applied for the calculation of the periodicity of such fibrils. The method is based on the periodic variations in intensity along the fibril[71]. Calculations were performed on skin collagen fibrils from mice, rats, and rabbits. Fibrils were without treatment, i.e., normal fibrils, or treated with lithium at various doses and for various experimental periods. No effects of lithium on the axial periodicity of skin collagen are detected. Similar results are obtained in rat and rabbit liver as well as in rat aorta collagen fibrils.

## CONCLUDING REMARKS

This review demonstrates that lithium induces structural abnormalities in collagen fibrils in a variety of specimens — skin, liver, bone, arachnoid and dura mater, as well as in the thoracic descending aorta — from different species: mice, rats, and rabbits. These abnormalities, which are summarized in Table 1, consist of decreased mean diameter and irregularity of shape of collagen fibrils (when viewed in cross-sections) as well as marked disorganization in the packing of the fibrils. It is important to recognize that these abnormal structures are present in the studied tissues after a relatively short period of treatment

**Table 1: Effects of lithium on collagen fibril structure from various tissues**

| Type of tissue | | | Packing of fibrils | Cross-sections | Mean fibril diameter |
|---|---|---|---|---|---|
| | Lithium dose | Lithium duration | Extent of disorganization | Extent of shape irregularity | Severity of alteration |
| Skin | 0.7–6.0 meq Li/kg | 30 days | ++++ | ++++ | ++++ |
| | 0.3 meq Li/kg | 30 days | + | | + |
| | 0.7–1.5 meq Li/kg | 7 days | +++ | | ++ |
| | 0.3 meq Li/kg | 7 days | + | | |
| Liver | 1.5 meq Li/k | 30 days | +++ | ++ | ++++ |
| Bone | 1.5 meq Li/kg | 30 days | ++ | + | ++ |
| Brain | 1.5 meq Li/kg | 30 days | ++++ | ++++ | ++++ |
| Aorta | 1.5 meq Li/kg | 30 days | ++++ | | ++ |

(from 7 to 30 days). This was also observed by McAuliffe and Olesen[72], who pointed out that short periods (7 weeks) of oral lithium therapy can cause significant changes in the structure of various portions of the nephron in rats. Even when the dose is maintained close or below the therapeutic limits (0.7 or 0.3 meq Li/kg) at very short treatment periods, 7 days, fibril disorganization is the persistent observation while fibril diameter is less affected, indicating that this parameter is dependent on the duration of lithium treatment. Nevertheless, the possibility as to whether or not any collagen structural changes would be further enhanced if lithium is taken for a prolonged period of time needs to be considered.

In contrast to bone and aorta, in skin, liver, and arachnoid and dura mater collagen, alterations induced by lithium are detected from the early stage after lithium treatment, i.e., 1 day. In the first two tissues, they become prominent at prolonged time after treatment. Lines of evidence suggest that collagen fibril diameter is related to location within the tissue[73]. Other data point to a link between collagen fibril diameter and mechanical properties of the specific tissue[74,75]. Thus, lithium could affect collagen involved in different tissues by a different way.

A point worth noting is that even at prolonged times after lithium treatment, 6 and 12 months, collagen abnormalities are more severe than at earlier stages, 1 day. This occurs in all tissues studied. It is not clear why lithium effects become more dramatic after a prolonged time after treatment. In arachnoid and dura mater, type I collagen is the major type in association with minor amounts of type IV collagen[76,77]; also, the predominance of type I collagen in skin during development suggests that this collagen by itself may play an important role in the organization of fibrils in these two tissues.

In skin, liver, bone, and aorta, type I is associated with type III collagen, which means that the collagen fibrils from these tissues are heterotypic. Among other factors, the interactions of type I and III collagen molecules, as well as the amounts of these collagen molecules relative to one another, may be important for regulation of fibril structure. Waterhouse et al.[41] pointed out that lithium does not affect the types of collagen synthesized by murine bone marrow cells, although the relative proportions of collagen types may differ from controls. There is evidence from the work of Adachi et al.[78] and Imamura et al.[79] that collagen subtypes, V and pNcollagen III, present in the above tissues are considered to have a crucial role in determining the diameter of heterotypic collagen fibrils. Much remains to be investigated about the factors controlling collagen fibril diameter on lithium treatment and their relationship to the structural disorganization of fibrils caused by lithium.

The abnormal appearance of bundles of collagen fibrils after lithium treatment may not be due to changes in the collagen molecule itself, but could arise from alterations occurring in other components of the extracellular matrix. Smaller proteoglycans are known to play an important interfibrillar role and lithium-induced alterations to these proteoglycans could have a profound effect on the morphology of collagen fibril bundles.

The pathogenic mechanism responsible for these abnormalities is still obscure. Alterations could occur at any level of collagen biosynthesis and extracellular processing. Tyobeka and Becker[80] reported that the plasma membrane is the main target for lithium cytotoxicity, while cytotoxicity might be related to the accumulation of lithium within the cells. Whether or not lithium affects collagen in the intracellular or extracellular process requires further studies. Also, the possibility that lithium may influence the rate of collagen synthesis and degradation should be taken into consideration. Collagen presents a turnover that greatly depends on the age and tissue of the animal studied[81]. Collagenase, an enzyme known to exist bound to collagen fibrils, plays an important role in this turnover[82]. Regulatory mechanisms that may affect the activity of this enzyme on lithium treatment may be elucidated by further investigations.

Results of the collagen fibril periodicity in lithium-treated and -untreated tissues, as measured with a computer-aided method, point to no shrinkage of the treated tissues in contrast with other treatments[83]. However, rupture of the rat aorta is influenced by lithium. Under lithium administration for 30 consecutive days, breaking pressure is significantly lower than that obtained without treatment[64]. Groenink et al.[84] demonstrated a correlation between aortic stiffness and breaking stress of the human thoracic aorta. Collagen and elastin are crucial in determining the tensile strength and the stiffness of the aorta[85]. Also, extracellular matrix components other than collagen and elastin, such as proteoglycans, may modulate vascular stiffness. We postulate that a structural defect in any one or more of these components induced by lithium increases the load bearing on the other components of the aortic wall and, thus, makes the vascular wall more susceptible to failure. Furthermore, it is reported that in unipolar- and bipolar-depressed patients, the prescription of lithium was associated with significant increases in blood pressure[86]. This may be a rationale for further studies concerning patients with aortic disease who are under lithium therapy.

In conclusion, lithium seems to induce permanent structural collagen alterations. Even at 12 months after the end of treatment, collagen fibril structure does not return to normal. These observations may indicate that collagen might be a valuable model for understanding the side effects of lithium on various organ systems of the body and exploring its mechanism of action. To maximize possible lithium effects on collagen structure, it is necessary to use as high a dose as possible. For this reason, the dose used in most cases in the work described in this review is greater than that used most commonly in psychiatric patients. Therefore, we do not suggest that these results may be directly applicable to human patients receiving long-term lithium for recurrent affective disorders. The obtained results may be a toxic effect. Lithium has substantial toxic effects at relatively small increments to normal therapeutic doses and although it has been used for over 50 years, toxicity still occurs frequently[87].

## BIOSKETCH

Awarded the Alexandros Onasis Fellowship, Professor Tzaphlidou received her PhD in Medical Biophysics from the University of Manchester, U.K., in 1980. After fellowships awarded by the British Council, Professor Tzaphlidou was awarded a senior fellowship with NATO and has been a project evaluator for the European Commission, the Greek Ministry of Research and Technology, the Greek Ministry of Education, and the Italian Ministry for Education, University and Research.

In 1981, Professor Tzaphlidou established the Biophysics Laboratory in the Department of Medical Physics, Ioannina University, which has produced some 60 publications and contributed to over 80 conferences. The activities of the Lab cover a wide variety of subjects related to research and development:

- Health Sciences: connective tissues in health and disease, drugs action, electron microscopy, image analysis, protein modeling, body composition studies, trace/major elements
- Electromagnetism: biological effects, dosimetry, electronics
- Medical Engineering: electronics in medicine, medical biotechnology
- Informatics: databases, data mining, human network
- International Education

Since 1998, Professor Tzaphlidou has been involved in the activities of the UNESCO Chair in Life Science - Life Sciences International Higher Educational Schools (LSIHES).

In addition to her research interests, current responsibilities include the organization and teaching of Medical Physics and Biophysics to medical students, supervision of PhD students, and as a visiting lecturer to universities around the world.

Professor Tzaphlidou brings much experience as editor and board member of International Scientific Journals, including as Guest Editor of three special issues for the international journal *Micron*.
